# Maintenance of recovered dilated cardiomyopathy patients with half-dose neurohumoral blockades (MED-CHARM): A protocol for an open-label, pilot, randomized trial

**DOI:** 10.3389/fcvm.2022.966537

**Published:** 2022-08-12

**Authors:** Pengda Li, Xiaolin Luo, Changchun Hou, Shaofa Wu, Luyu Wang, Ning Sun, Zebi Wang, Zelan Wang, Jun Jin, Jiang Wang, Zhexue Qin

**Affiliations:** ^1^Department of Cardiology, Xinqiao Hospital, Army Medical University (Third Military Medical University), Chongqing, China; ^2^Xinqiao Hospital, Institute of Cardiovascular Diseases of PLA, Army Medical University (Third Military Medical University), Chongqing, China

**Keywords:** recovered dilated cardiomyopathy, neurohumoral blockades, heart failure, dosage adjustment, randomized controlled trail

## Abstract

Dilated cardiomyopathy (DCM) has brought great damage to the patients' health and social economy. The number of patients with recovered dilated cardiomyopathy (recDCM) has increased over the years as treatment progresses. However, there is a lack of relevant evidence to support the clinical management of patients with recDCM, thereby, the recommendations in guidelines remains sparse. Accordingly, the exploration of recDCM is important to improve patient prognosis and reduce societal burden. This is an open-label, randomized controlled, prospective study that will compare the safety and efficacy of original dose and halved dose of neurohumoral blockades for patients with recDCM.

**Methods:**

An open-label, randomized controlled, prospective study will be conducted among eligible patients with recDCM. During the pilot study phase, we will recruit 50 patients. The primary endpoint is hospitalization for heart failure or heart failure relapse within 12 months. Secondary endpoint is major adverse cardiovascular events, including cardiovascular mortality, myocardial infarction, stroke, sustained atrial tachycardia, or ventricular tachycardia. The results will be analyzed using intention-to-treatment analysis.

**Discussion:**

The study will provide important evidence of whether it is safe and effective to halve the dosage of neurohumoral blockades in recDCM patients.

**Trial registration number:**

ChiCTR2100054051 (www.chictr.org.cn)

## Background and rationale

Patients with dilated cardiomyopathy (DCM) have a poor prognosis, manifested by persistent left ventricular enlargement and progressive deterioration of systolic function, ultimately leading to heart transplantation or death (1). On the other hand, the disease burden of DCM is increasing globally, including in China. In 2015, the global burden of disease study estimated that the global number of cardiomyopathy is 2.5 million, a figure that increased by 27% from 10 years ago (2). A study in China in 2014 showed a mortality of 42.24% among 767 patients with DCM in 52 months (3). Furthermore, to the best of our knowledge, DCM often affects young patients with fewer comorbidities and a theoretically high life expectancy (4). Consequently, the exploration of the progress and outcome in patients with DCM is important to improve the prognosis and reduce societal burden.

DCM patients with heart failure have improved survival with the benefits of angiotensin-converting enzyme inhibitors (ACEI) or angiotensin receptor blockers (ARB), beta-blockers, and spironolactone (5). The complete normalization of left ventricular ejection fraction (LVEF) in some heart failure patients has been termed heart failure with recovered ejection fraction (HFrecEF) (6). Among patients with HFrecEF, DCM occupies a high proportion, just second to heart failure resulting from energy and metabolic disorders (e.g., thyroid disease-related cardiomyopathy) (6). Due to the lack of a standardized definition and the current data from observational studies and clinical trials, the proportion of patients with improved ejection fraction ranges from 10% to 40% (6). In some of the patients with DCM, the ejection fraction and cardiac chamber size was observed to completely return to normal, which has been termed recovered dilated cardiomyopathy (recDCM) in some studies (7). However, there is a lack of relevant evidence to support the clinical management of patients with recDCM, thereby, the recommendations in guidelines remains sparse.

The fact that there has only been one prior prospective, randomized, controlled study in patients with recDCM is a mixed blessing. The TRED-HF study, withdrawal of pharmacological treatment for heart failure in patients with recovered dilated cardiomyopathy, published in *Lancet* in 2018, explored the safety and efficacy of withdrawing all drugs in patients with recDCM. That study found a significantly higher proportion of heart failure recurrence in the withdrawn group, while in the control group, there was no relapse at half-year follow-up, indicating that such patients were not suitable for total withdrawal treatment (7). However, all the patients in the control group took the guideline-recommended dosage, and it is not clear whether the cardiac function could be maintained normal if the drug class is not changed while the dose is halved.

In addition, many previous studies point out that in the actual clinical practice, many heart failure patients often fail to reach the target doses of guideline-directed medical therapy (GDMT) because of complicated multiple comorbidities (8–12), which is a great challenge to successfully up-titrate classes of HF medications (13). Moreover, a previous study showed that the best outcomes among heart failure patients were observed in those with a combination of ACEI/ARB and beta-blockers therapy, and unfortunately, this was rarely reached. Patients with the combination therapy of the two drugs reaching more than half of the target dose have better prognosis than those with a single drug therapy titrating to the target dose (14). Accordingly, we assume that the above speculation may apply to the clinical practice of recDCM.

Insights into the pathophysiological mechanisms of heart failure in recovered DCM patients, as well as advances in drugs for heart failure, have enabled the possibility of drug reduction in such patients. First, factors promoting adverse cardiac remodeling in patients with heart failure with recovered ejection fraction have been already suppressed with guideline-directed medications, and one study suggests that there may be a strong attenuation of adverse remodeling factors, such as sympathetic activation and renin angiotensin aldosterone system (RAAS) activation (6). In such circumstance, it may be possible for recDCM patients to reduce the doses of medications for heart failure. Second, with the advent of angiotensin receptor-neprilysin inhibitor (ARNI) and sodium-glucose cotransporter 2 inhibitors (SGLT2-i), patients have experienced significant improvement in the treatment of heart failure compared with previous ones (15). As a consequence, with the application of current novel class of drugs inhibiting cardiac remodeling, the original “ACEI/ARB/ARNI + beta-blockers” doses may have room for diminution. Actually, there are instances in which cardiac function is normalized in a subset of patients with low doses of GDMT for heart failure (14). These indicate that there may be a subset of patients who do not require a target dose to maintain cardiac function, and lower doses may be adequate. Thus, we reasoned that the “ACEI/ARB/ARNI + beta-blockers” halving dose therapy may be non-inferior to original target dose in maintaining cardiac function in recDCM patients.

This is an open-label, randomized controlled, prospective study that will be conducted in patients with recDCM, comparing the safety and efficacy of original target dose and halved dose of “ACEI/ARB/ARNI + beta-blockers.” This will provide essential evidence of whether it is safe and effective to reduce doses of neurohumoral blockades in recDCM patients.

## Methods and analysis

This is an open-label, randomized controlled, prospective study. Following the guidance of the ethical committee at our institution, we will recruit 50 patients in the pilot study phase.

### Patients' recruitment

All patients with recDCM referred to Xinqiao Hospital (Army Medical University, Chongqing, China) will be screened by a senior cardiologist. Patients and their guardians are required to sign written informed consent. A special staff member will carefully describe the study to the patients and their guardians.

### Inclusion criteria

1. Age≥18 years old.
2. Previous diagnosis of dilated cardiomyopathy with LVEF <40%.
3. Current therapeutic drugs with at least one of ACEI/ARB/ARNI and beta-blockers. Both ACEI/ARB/ARNI and beta-blockers doses are not up-titrated within 6 months; Other GDMT include a mineralocorticoid receptor antagonist (MRA), a SGLT2-i or diuretics.
4. Without symptoms of heart failure, and no hospitalization for heart failure for more than 6 months.
5. With at least two independent echocardiography (with interval ≥6 months) show that LVEF ≥50%, and left ventricular end-diastolic volume index (LVEDVi) <97ml/m^2^.
6. With written informed consent.

### Exclusion criteria

One of the following conditions is met:

1. Uncontrolled hypertension (blood pressure≥160/100 mmHg).
2. Valvular heart disease with moderate or greater severity.
3. Severe renal insufficiency (estimated glomerular filtration rate (eGFR) <30 ml/min/1.73 m^2^, estimated according to Cockcroft-Gault formula).
4. Atrial, supra-ventricular, or ventricular arrhythmia requiring beta-blockers.
5. With the implantation of intra-cardiac defibrillator or cardiac resynchronization therapy.
6. Ischemic heart disease.
7. With severe systemic diseases.
8. Diagnosed with secondary cardiomyopathies.

### Randomization

The enrolled patients will be randomly assigned into two groups. The randomization list will be automatically generated by the computer system. Before the start of the trial, the randomization list will be configured into the interactive network response system (IWRS, Jinling Rat mini-apps, Nanjing Jihu Network Technology Co., Ltd, Jiangsu, China), and IWRS will assign random numbers to these finally screened patients. Finally, the patients will be randomly assigned to either the withdraw group or control group with a ratio of 1:1 using IWRS.

### Primary endpoint

The patients will be followed up for 12 months. The primary endpoint is hospitalization for heart failure or heart failure relapse. Heart failure relapse is defined if one of the following criteria is met: (1) LVEF reduction≥10% with LVEF <50%; (2) LVEDVi increased by more than 10%, and exceeded the normal values; (3) 2-fold elevation of NT-proBNP and more than 400 pg/ml; (4) Clinical evidence of heart failure (judged by symptoms, signs and supplementary examination).

### Secondary endpoint

Secondary endpoints are major adverse cardiovascular events, including cardiovascular mortality, myocardial infarction, stroke, sustained atrial tachycardia, or ventricular tachycardia.

### Sample size

Regarding the results from clinical trials in heart failure patients, the incidence of cardiac death or heart failure readmission within 12 months is 15–30% with guideline-directed medical therapy. The patients with DCM enrolled in this study have recovered cardiac function and ejection fraction, and TRED-HF study showed a primary endpoint event rate of 0% in such patients with medication doses unchanged in 6 months. Thus, in this study, it is estimated that the primary endpoint rate in control group and in withdraw group is 5 and 7% over 12 months of follow-up. It is estimated that 218 patients would provide the trial with 80% power to show the non-inferiority of halved doses to original doses, a two-sided alpha of 0.05 and a non-inferiority margin of 10% is applied. Considering a loss to follow up rate of 10%, the final sample size is 240. According to the suggestion of the ethical committee, the current study is a pilot study and 50 patients are intended to be included initially, 25 patients per group. After the initial analysis, more patients might be recruited in a further stage of the study.

### Statistical analysis

Studies will be analyzed using intention-to-treatment and included all patients randomized, regardless of treatment received. Continuous data are expressed as mean ± SD or interquartile range (IQR 25–75). Categorical variables are expressed as a percentage. Continuous variables are compared by an independent sample *t*-test or rank-sum test. The chi-square or Fisher's exact test is used in the analysis of categorical variables. The Cox proportional hazards model was used to analyze and compare the differences in the occurrence of primary endpoint events and key secondary endpoint events within 12 months of follow-up between the trial group and the control group, and the hazard ratio (HR) and 95 % confidence interval will be calculated.

### Intervention

**Figure 1:**
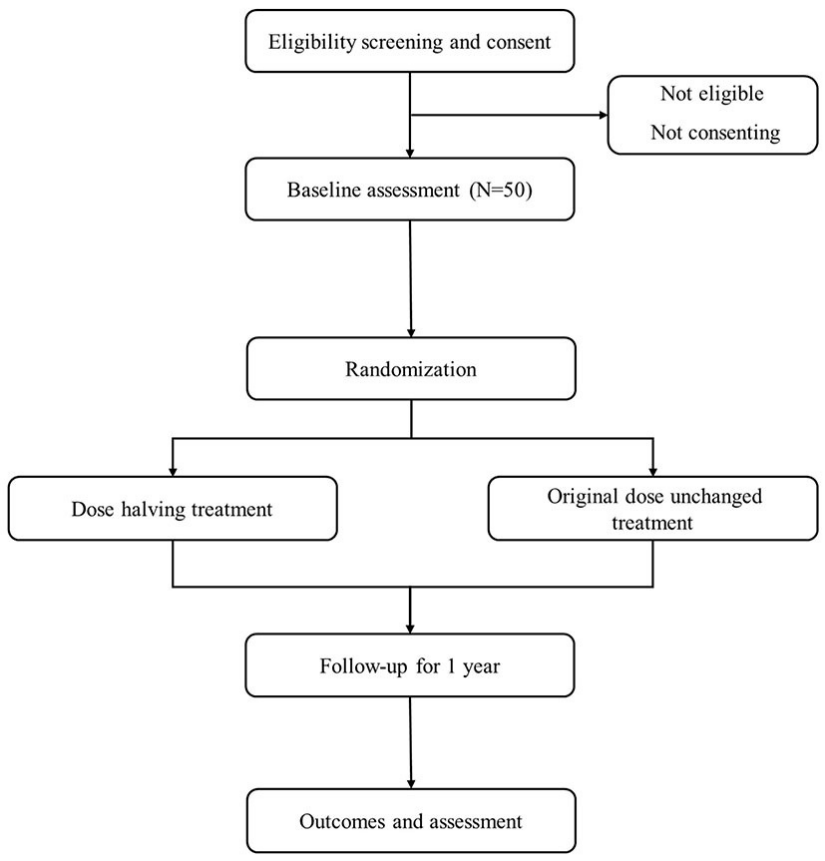
Study flow diagram.

According to ICH-GCP and local regulations, no study procedure can be started until the patient signs the written informed consent. The screening will be carried out after accomplishing the patient's written informed consent. The flow chart is shown in Figure 1. The patients who only meet research criteria will be randomized, otherwise, they will be not.

In the withdraw group, the doses of ACEI/ARB/ARNI will be reduced by 50% firstly. The symptoms and signs will be assessed in the following 1 month. The echocardiography and NT-proBNP will be performed to see if they could tolerate a further dose reduction of beta-blockers. Then, the dose of beta-blockers will also be reduced by 50% if the cardiac function of participants is stable. The other drugs of the participants will keep unchanged during the follow-up. In the control group, ACEI/ARB/ARNI, beta-blockers, spironolactone, SGLT2-i, and other drugs will be taken with original doses during the follow up.

### Follow-up and measurement

The participants in this study will be followed up by face-to-face interviews and telephone. First, the demographic data, medical history, physical examination, laboratory tests will be documented as baseline at the beginning of the study. Then, the cardiac function (NYHA classification), 6-min walking test, and Kansas City Cardiomyopathy Questionnaire (KCCQ) score will be evaluated. Patients will be followed up at 1st, 3rd, 6th, and 12th month. Hospitalization for heart failure, heart failure relapse, and other adverse events will be documented. In the last follow-up, the participants will accomplish the examinations such as genes and metabolites and cardiac MRI as appropriate according to the evaluation.

### Patient and public involvement

Patients, their guardians, and public representatives will be informed about the study, and yet they were neither involved development of study questions nor the planning of research design. In the same way, they also neither took part in the recruitment nor the conduct of this study. Results of the study will be published only in peer-reviewed journals, it is no other information on the results of the study that are provided to patients and their guardians.

### Ethics and dissemination

The study protocol has been approved by an ethical committee of Xinqiao hospital, The Army Medical University, Chongqing, China, and was registered at the Chinese Clinical Trial Register (www.chictr.org.cn, ChiCTR2100054051). Patients and their guardians are required to sign written informed consent if the inclusion criteria are met before the beginning of the study, and meanwhile, researchers are supposed to be sure of participants' voluntariness strictly. When there are clear and ongoing contraindications or the patient requests that the study be terminated, the study should be terminated as soon as possible. Participants are free to withdraw at any time. Important modifications to the protocol that may affect the study's progress will be reported to the above-mentioned committee. Results of the study will be disseminated as published articles in peer-reviewed journals.

### Schedule

The participants will be registered from December 1, 2021, to December 31, 2023. The end date of follow-up is December 31, 2024.

## Discussion

The main aim of the present study is to evaluate the efficacy and safety of patients with recDCM who received half-dose neurohumoral blockades and received original dose maintenance therapy, and then by comparing the two doses of treatment, it may be concluded that “ACEI/ARB/ARNI + beta-blockers” halving dose was non-inferior to patients' maintenance dose in maintaining recovered cardiac function in DCM patients. This will provide a significant basis for the choice of whether to reduce dosage or to adhere to original dose maintenance therapy in DCM patients who recovered, to reduce the economic and psychological burden on patients.

Furthermore, this study is only the pilot study of the original research we want to do because the ethics committee needs to protect the interests of patients. Therefore, one of the limitations of this study is the small sample size, and if this study is effective, we will conduct more research.

## Data availability statement

The datasets during analyzed and/or during the current study are available from the corresponding author upon reasonable request.

## Ethics statement

The studies involving human participants were reviewed and approved by Medical Ethics Committee Office, Pharmacology Building, No. 183 Xinqiao zheng Street, Shapinba District, Chongqing. The patients/participants provided their written informed consent to participate in this study.

## Author contributions

ZQ, XL, JW, and JJ designed the study. PL and XL drafted the manuscript. PL, CH, SW, LW, and NS helped to critically revised the drafts of the manuscript. ZebW and ZelW read and approved the final manuscript. All authors contributed to the article and approved the submitted version.

## Funding

This study was supported by the Chongqing Medical Scientific Research Project (Joint Project of Chongqing Health Commission and Science and Technology Bureau) (No. 2022MSXM115).

## Conflict of interest

The authors declare that the research was conducted in the absence of any commercial or financial relationships that could be construed as a potential conflict of interest.

## Publisher's note

All claims expressed in this article are solely those of the authors and do not necessarily represent those of their affiliated organizations, or those of the publisher, the editors and the reviewers. Any product that may be evaluated in this article, or claim that may be made by its manufacturer, is not guaranteed or endorsed by the publisher.
